# Achieving behaviour change at scale: Alive & Thrive's infant and young child feeding programme in Bangladesh

**DOI:** 10.1111/mcn.12277

**Published:** 2016-05-17

**Authors:** Tina Sanghvi, Raisul Haque, Sumitro Roy, Kaosar Afsana, Renata Seidel, Sanjeeda Islam, Ann Jimerson, Jean Baker

**Affiliations:** ^1^ FHI 360 Washington District of Columbia USA; ^2^ BRAC Dhaka Bangladesh

**Keywords:** scaling up nutrition, behaviour change, hygiene improvement, breastfeeding, complementary feeding, Bangladesh

## Abstract

**Abstract:**

The Alive & Thrive programme scaled up infant and young child feeding interventions in Bangladesh from 2010 to 2014. In all, 8.5 million mothers benefited. Approaches – including improved counselling by frontline health workers during home visits; community mobilization; mass media campaigns reaching mothers, fathers and opinion leaders; and policy advocacy – led to rapid and significant improvements in key practices related to breastfeeding and complementary feeding. (Evaluation results are forthcoming.) Intervention design was based on extensive formative research and behaviour change theory and principles and was tailored to the local context. The programme focused on small, achievable actions for key audience segments identified through rigorous testing. Promotion strategies took into account underlying behavioural determinants and reached a high per cent of the priority groups through repeated contacts. Community volunteers received monetary incentives for mothers in their areas who practised recommended behaviours. Programme monitoring, midterm surveys and additional small studies to answer questions led to ongoing adjustments. Scale‐up was achieved through streamlining of tools and strategies, government branding, phased expansion through BRAC – a local non‐governmental implementing partner with an extensive community‐based platform – and nationwide mainstreaming through multiple non‐governmental organization and government programmes.

**Key messages**

- Well‐designed and well‐implemented large‐scale interventions that combine interpersonal counselling, community mobilization, advocacy, mass communication and strategic use of data have great potential to improve IYCF practices rapidly.
- Formative research and ongoing studies are essential to tailor strategies to the local context and to the perspectives of mothers, family members, influential community members and policymakers. Continued use of data to adjust programme elements is also central to the process.
- Scale‐up can be facilitated through strategic selection of partners with existing community‐based platforms and through mass media, where a high proportion of the target audience can be reached through communication channels such as broadcast media.
- Sustaining the impacts will involve commitments from government and capacity building. The next step for capacity building would involve understanding barriers and constraints and then coming up with appropriate strategies to address them. One of the limitations we experienced was rapid transition of staff in key positions of implementing agencies, in government leadership, donors and other stakeholders. There was a need for continued advocacy, orientation and teaching related to strategic programme design, behaviour change, effective implementation and use of data.

## Introduction

Direct nutrition interventions such as improving infant and young child feeding (IYCF) practices are considered a high priority for programmes aimed at improving health and nutritional status and child survival (Dewey & Brown 2003; Lutter *et al*. 2011; Onyango 2013). Breastfeeding is one of the most cost‐effective life‐saving interventions known (Bhutta *et al*. 2008). Stewart *et al*. (2013) highlighted the role of complementary feeding in reducing stunting. Bhutta *et al*. (2013) estimated that more than 220 000 lives would be saved each year with delivery of an infant and young child nutrition package that includes breastfeeding and complementary feeding. In Bangladesh, scaling up these interventions would benefit, in terms of child health and mortality, the two poorest wealth quintiles more than twice as much as upper quintiles, suggesting an important effect on reducing health and economic disparities (Bhutta *et al*. 2013).

According to the World Health Organization (WHO), in many countries, less than a fourth of infants 6–23 months of age receive the WHO‐recommended amounts and variety of foods, including breast milk (WHO 2015). Stewart *et al*. (2013) emphasized the complex web of community and societal factors that influence decisions about IYCF. Barriers may include poor food availability and purchasing power, knowledge gaps, social norms or a combination of these. Programmes need to address multiple factors (Lutter *et al*. 2013).

Effective IYCF interventions have included the following: (1) time‐targeted interpersonal counselling, individually and in groups, provided by trained health care professionals or lay workers; (2) assistance to pregnant and newly delivered mothers in initiating lactation immediately after delivery and in continuing breastfeeding; (3) provision of food and related supplements for complementary feeding; (4) media and marketing approaches; and (5) legislation to support restriction of the indiscriminate marketing of breast milk substitutes and parental leave to enable mothers to breastfeed exclusively for 6 months (Caulfield *et al*. 1999; Merten *et al*. 2005; Bhutta *et al*. 2008; Dewey & Adu‐Afarwuah 2008; Imdad *et al*. 2011; Wakefield *et al*. 2011; Lutter & Lutter 2012).

A number of countries, including Bangladesh, have incorporated recommendations from WHO's *Global Strategy for Infant and Young Child Feeding* (2003) in their national strategies. Scaling up proven interventions has been a challenge, however.

The Bill & Melinda Gates Foundation designed the Alive & Thrive (A&T) initiative to develop scale models for IYCF in three distinct geographies: Bangladesh, Ethiopia and Vietnam. In this paper, we document the process of scaling up an evidence‐based IYCF intervention in Bangladesh. Results of quantitative studies documenting significant changes in IYCF practices in the A&T programme area are forthcoming. Information on the A&T programmes in Ethiopia and Vietnam is available at http://www.aliveandthrive.org. See also Baker *et al*. (2013).

## Materials and methods

**Figure 1:**
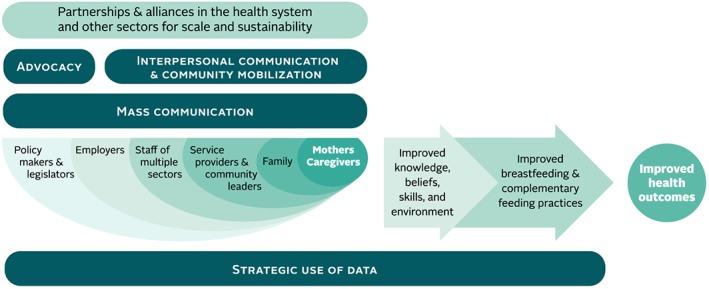
Framework for delivering nutrition behaviour change results at scale.

In Bangladesh, the programme aimed to develop a streamlined and easily scaled‐up package of activities. A&T partnered initially with the Essential Health Care (EHC) programme of BRAC, a respected national non‐governmental organization (NGO) operating large community‐based public health, education and microfinance programmes. Programme design involved four components: advocacy, interpersonal counselling and community mobilization, mass communication and the strategic use of data (Box 1 and Fig. 1). All strategic decisions were evidence based. After programme launch, ongoing data from programme monitoring, assessments, small studies and quantitative surveys were used to adjust strategies. The programme thus evolved during the process of scale‐up. Summarized thereafter are the categories and timing of research conducted.

**Box 1. A&T programme components (excluding research)**

Advocacy to promote child nutrition and accelerate scale‐up of programmes:

- Local, regional and national decision makers and stakeholders: Advocacy video shows on IYCF, meetings with the national alliance of over 20 stakeholders under the Institute of Public Health Nutrition (IPHN)/Government of Bangladesh (GOB), dissemination of government‐branded materials to implementing stakeholders.
- Engaging journalists: Orientations and scholarships for journalists, TV talk shows and newspaper supplements.
- Individualized dialogue with government decision makers and donors: MOUs, task forces and sharing evidence.

Interpersonal counselling and community mobilization

- Mothers: Counselling provided through home visits at specific ages of the child on breastfeeding, complementary feeding and handwashing before feeding, and group community meetings of pregnant and lactating women with trained community workers.
- Community opinion leaders: Mobilization of support for child nutrition and IYCF through orientations, video shows, seminars and forums to reach doctors, religious leaders, fathers, local government and NGOs working at community level.
- Health providers: 3‐ to 5‐day in‐service training, pre‐service medical/nursing curriculum through partnerships with medical associations, mass media and print materials, messages through national and regional newspapers, wall posters for government and private clinics, job aids for government staff and materials tailored for formal and informal health practitioners.

Mass media

- Families, frontline workers and opinion leaders: TV and radio spots on key topics for mothers, fathers, frontline workers and opinion leaders at all levels.
- Rural community members in media dark areas: Interactive community events including village theatre, community video showings and quiz shows on IYCF and handwashing topics.

### Situation analysis and first round of formative research – 2009

Alive & Thrive's situational analysis included a review of policies and strategy guidelines of the GOB, data on media habits, case studies and relevant research (Haider *et al*. 2000; Kimmons *et al*. 2005; Saha *et al*. 2008; Rasheed *et al*. 2009) and further analyses of IYCF and household indicators in the two most recent Bangladesh Demographic and Health Surveys (NIPORT 2005; NIPORT 2009).

Formative research tools were adapted from a process developed by the Pan American Health Organization called ProPAN (PAHO 2004). These studies are described in Haider *et al*. (2010) and Rasheed *et al*. (2011), and the results have already been published in these articles. They were carried out from May 2009 to October 2009 in collaboration with the International Centre for Diarrhoeal Disease Research, Bangladesh, in three areas with high malnutrition: Sylhet, Chittagong and Dhaka slums. Research focused on mothers' perceptions and practices, media habits and reach, health worker practices and work environment, and promotion of breast milk substitutes at the retail level and through the media. Table 1 lists the methods and organizations responsible for the research. A&T selected priority nutrition practices for programme focus (Box 2), followed by rapid trials of practices with mothers to the test feasibility of introducing small changes into current behaviours.

**Table:** Box 2. Programme target behaviours – major indicators*

1. Early initiation of breastfeeding (within 1 h)
2. Exclusive breastfeeding under 6 months of age
3. Continued breastfeeding at 1 year
4. Timely introduction of complementary foods (6–8 months)
5. Minimum dietary diversity
6. Minimum meal frequency
7. Minimum acceptable diet
8. Consumption of iron‐rich foods

* WHO definition. See WHO 2008.

**Table 1:** Formative research components

Methods	Topics covered	Respondents and data sources
Formative research at start‐up		
Household survey	Practices and access to services/media	*N* = 358 mothers of children aged 0–23 months
24‐h dietary recall	Dietary intake and complementary feeding	*N* = 197 children aged 6–23 months
Rapid trials of practices	Barriers, motivations and drivers of behaviour change	*N* = 119 mothers of children aged 0–18 months
Market survey	Food sources and prices	Retail outlets in three sites
Semi‐structured interviews	Reasons for IYCF and main influences	*N* = 42 mothers of children 0–23 months
Food attributes exercise	Perceptions about foods	*N* = 23 mothers of children 6–23 months
Opportunistic observations	Perceptions and practices	*N* = 21 (traditional birth attendants and village doctors)
Media scan (Nielsen)	Media habits (secondary data)	Demographic and Health Surveys (DHS) and Bangladesh Media and Demographic Survey 2008
Additional analysis of Demographic and Health Survey (2007)	IYCF practices and use of animal source foods (secondary data)	*N* = 2858 mothers of children aged 0–35 months
Health care workers (BRAC)	Perceptions	*N* = 69 (formal and informal providers)
Baseline survey (IFPRI)	Household survey	*N* = 2000 mothers of children <24 months
Ongoing research		
Doctors survey	KAP of IYCF and media habits	*N* = 150 doctors in five regions
Handwashing formative research (ICDDR,B)	Barriers, motivations and rapid trial	Quantitative (*n* = 300), qualitative (*n* = 25) and trials of practices (*n* = 80 mothers of children aged 6–23 months)
Programme monitoring	Home visits and media reach	*N* = variable
Midline surveys (IFPRI)	IYCF, coverage and perceptions	*N* = 453 children aged 6–23 months (2012)
		*N* = 2000 children aged <24 months (2013)

ICDDR,B, International Centre for Diarrhoeal Disease Research, Bangladesh; IFPRI, International Food Policy Research Institute; IYCF, infant and young child feeding; KAP, knowledge, attitudes and practices.

**Box 3. Programme evolution/scale‐up milestones**

- Field test of community strategies in four BRAC programmes: September 2009 to January 2010
- GOB national alliance formation and issuing of a national communication plan: December 2009 to March 2010 (the alliance continues)
- Community component launched in BRAC EHC programme in 22 sub‐districts: August to December 2010
- Media campaign launch and intensification: December 2010 to March 2011
- Campaign materials on handwashing before feeding developed (based on additional formative research): April 2011 to July 2012
- Community component reaches 50 sub‐districts in BRAC's EHC programme: August 2011
- Final field testing and revisions of training module and job aids, rebranded as GOB materials and disseminated nationally: January 2011 to November 2011
- Interpersonal counselling scaled up through national government providers and NGO maternal newborn and child health and DFID‐funded livelihoods programmes: August 2011 to December 2013
- Media dark strategy designed and tested and community events launched in targeted low reach villages: February 2012 to June 2014
- Media campaign developed and print materials launched for handwashing before feeding: October 2012
- Media campaign for doctors developed and launched: January 2012
- Interpersonal counselling scaled up through GOB Health and Family Welfare workers: March 2013 to mid‐2014

### Baseline survey – 2010

Alive & Thrive partner International Food Policy Research Institute (IFPRI) conducted a baseline household survey in 2010 with a sample of 2000 households having children below 24 months of age. The study was carried out in 20 sub‐districts from five of six regions in Bangladesh. Questions focused on the WHO‐recommended IYCF practices; household, maternal and child characteristics; and mothers' knowledge, contacts with health workers and media habits.

### Second round of formative research to design handwashing interventions – 2011 to 2012

After programme launch, A&T collaborated with International Centre for Diarrhoeal Disease Research, Bangladesh, to conduct additional formative research exploring caregivers' knowledge and practices of handwashing before child feeding, as well as sources of information on handwashing and preferred channels for messages. Methods included a household survey (*N* = 300), video data collection on 12 caregivers of children with a mean age of 15 months, an exercise for ranking motivations for handwashing with six groups, 24 in‐depth interviews with caregivers of children with a mean age of 16 months and six focus group discussions. The household survey and qualitative studies were conducted in 50 rural villages in three purposively selected districts in different regions of the country (Manikganj District, Dinajpur and Chittagong). Results are reported in Nizame *et al*. (2013).

### Monitoring mechanisms and rapid small studies 2010–2014

Monitoring focused on both delivery of community interventions (especially household visits) and media reach and recall. Data regarding community interventions were collected through three channels: routine service registers maintained by BRAC including indicators on community mobilization, home visits and mothers' IYCF practices; sample surveys by BRAC's independent monitoring wing; and random sample home visits conducted by A&T focusing on BRAC home visits, community mobilization, IYCF practices and media recall.

From 2010 onwards, A&T hired media placement companies to track TV viewing habits, via devices embedded in homes, in a random sample of households as part of a routine industry survey that calculates media reach for advertising and marketing companies. Data were triangulated with the recall‐aided questions in the IFPRI and A&T surveys to estimate coverage and recall of the media messages.

Alive & Thrive carried out several small studies after programme launch to examine gaps highlighted by the baseline or that emerged during monitoring or to field test evolving strategies. Studies included a survey of doctors' knowledge, attitudes and practices related to providing IYCF support to mothers and their media habits (*N* = 150). Other research included a pilot test of communication strategies for media dark areas, tests of options for short duration training and in‐kind incentives and field trials for participatory community approaches in Sylhet and Habiganj.

### Midline evaluation household surveys – 2012 and 2013

International Food Policy Research Institute conducted two midline household surveys. The 2012 survey measured WHO standard indicators for complementary feeding practices, exposure to home visits (by two types of frontline workers) and recall of TV spots broadcast by the programme. The sample included 462 households having children 6–23 months of age selected randomly from 100 villages in 10 programme sub‐districts. The 2013 survey included infants below 6 months (as well as older children) and looked at all IYCF‐recommended WHO core indicators for breastfeeding and complementary feeding, plus the coverage indicators. The sample included 500 households from intensive 1Intensive areas = A&T introduced more frequent home visits by trained workers and community mobilization in addition to national mass media campaign for IYCF. Target‐based monitoring was also used in these areas; managers and supervisors of frontline workers were asked to ensure that the estimated numbers of eligible households (based on census data) were enlisted and the protocol of age‐specific home visits was completed. A second cadre of full‐time IYCF promoters was allocated at a ratio of one per 10 volunteers to ensure that if the volunteer could not make the scheduled visits, the IYCF promoter would fill the gap. Non‐intensive areas = Less frequent, shorter duration routine home visits were ongoing plus the national mass media campaign on IYCF. programme areas and 500 from non‐intensive areas in 20 sub‐districts.

### Database on scaling up – 2011 onwards

Implementation partners (members of the National IYCF Alliance) shared information quarterly on their community‐based and facility‐based IYCF programmes. GOB mapped the location of these stakeholders, and A&T assisted with maintaining a database on the cumulative number of health workers and trainers provided with training. Information was shared at Alliance meetings.

## Results

### Programme implications of situation analysis

The existence of supportive national policies on child nutrition in Bangladesh meant that investments were not needed in this area and advocacy should focus on stimulating and supporting implementation of existing policies and guidelines. A&T also saw an urgent need for harmonizing efforts of multiple implementing NGOs and donors working in different geographic areas of the country. There was a clear opportunity to form a national alliance under the GOB's IPHN to leverage a high level of interest in IYCF and strengthen sustainability. As part of scale‐up efforts, A&T developed a national training module for the government's frontline workers. Government staff were trained by national and master trainers who were in turn trained by A&T at three government training centres in Dhaka and at one NGO training site (Lamb Hospital).

Advocacy for, and evidence of the effectiveness of interpersonal counselling, had been strong over several years, but large‐scale coverage of support for mothers was very limited. A&T saw the need to introduce strategies going beyond government health services. BRAC was the only NGO with scaled‐up presence in the country and had a ready‐made cadre of multi‐purpose community volunteers. They were a natural partner for introduction of new strategies.

Prior to 2009, promotion of breastfeeding had received far more emphasis in Bangladesh than promotion of optimal complementary feeding practices. While there was public awareness about the importance of exclusive breastfeeding for the first 6 months, practice was still not widespread. A&T therefore decided to focus on converting high awareness of breastfeeding to an emphasis on specific desirable practices and building a basic understanding about complementary feeding.

Secondary evidence clearly pointed to the importance and feasibility of promoting home‐based foods. Processed and/or fortified foods for children were not feasible as a nationally scalable strategy because of limited capacity for quality control and strong opposition from highly respected and influential IYCF advocates in the country. Household food insecurity was estimated to affect a quarter of the population, and three of four households were found to have animal source foods for child feeding (NIPORT 2009).

### Programme implications of formative research on IYCF practices

Formative research revealed that the major barrier to exclusive breastfeeding was mothers' perception of ‘insufficient milk’. A&T strategies therefore emphasized counselling on breastfeeding skills and simple remedies to prevent and overcome common difficulties (through correct positioning and attachment, manual expression of breast milk and frequent breastfeeding). Providers were trained to support mothers in accurately self‐assessing the adequacy of their breast milk production and in understanding how to maintain adequate breast milk supply.

Formative research also highlighted the importance of promoting animal source foods. Even when available in the home, these were not being given to young children.

The perception of poor appetite was widespread, leading mothers to lose confidence in proper complementary feeding. A&T messages discouraged giving children junk food, which was ubiquitous, and promoted simple strategies for motivating fussy eaters: encouraging food variety and age‐appropriate texture, reducing watery and low‐nutrient density snacks foods and spending more time feeding. Most mothers were available to spend time feeding children as outside employment is low and infant care is considered the mothers' main occupation, according to rural social norms.

For mothers, motivating factors for changes in IYCF practices included benefits for child's brain and physical development and safety or protection from illnesses, perception of positive child responses (‘child likes it’), convenience and satisfaction in being able to breastfeed adequately or motivate a child to eat enough quantity of complementary foods.

Alive & Thrive saw the importance and feasibility of developing a single national communication strategy. Results of the formative research and reviews of the DHS showed that patterns of good vs. weak IYCF practices were similar in urban and rural areas. Specifically, timely initiation, duration of exclusive breastfeeding and diversity of complementary foods were low in both urban and rural areas. The rapid trials of improved practices (TIPs) showed that mothers in urban and rural areas had similar reasons for common practices and similar motivations for adopting improved behaviours. Mass media reach and habits initially seemed largely the same, but more TV channels were available in urban areas. A&T decided to use mass media and community mobilization on a large scale. Secondary audiences who could encourage mothers (family members, health workers and neighbours) could also be reached rapidly through mass media.

### Programme implications of the media audit

The media audit provided evidence that TV should be the main channel for high reach/coverage at national scale. A loyal viewership (68% of households watching TV at least once a week), cutting across economic and education levels, and a single main language and cultural context were crucial factors. However, the audit also showed that media habits change rapidly, so media placements needed to be adjusted based on ongoing assessments. The media audit demonstrated that Bangladesh has effectively controlled mass media advertising of breast milk substitutes, but the situation needed ongoing monitoring.

### Programme implications of the 2010 baseline survey

Alive & Thrive's baseline survey confirmed the relative importance of different IYCF practices for programme emphasis. The survey also confirmed that even poor families consumed a diverse diet, including animal source foods, as well as vegetables and fruits – so that home‐based foods could be promoted as part of the IYCF strategy.

Mothers said that health professionals and family members were their primary sources of support when faced with problems with infant feeding. Food purchases were primarily the responsibility of male members of the family. This validated A&T's decision to target multiple influential audiences.

Although BRAC workers were found to be present in communities in many areas, baseline coverage of home visits to mothers was not very high, and there was substantial variability across sub‐districts. Overall, about a quarter of respondents had ever been visited at home by a BRAC frontline health volunteer (Shasthya Sebika). Contact between households and Shasthya Sebika went up to over 60% in some areas. A smaller proportion of respondents had been visited by a BRAC health worker (Shasthya Kormi) at 11.3%; 12% attended a health forum facilitated by a Shasthya Kormi.

BRAC's capacity to manage and organize mobilization of frontline workers on a large scale was strong, with great potential for strengthening home visits and community mobilization. BRAC decided to follow the example of their community‐based programmes (TB Dots; oral rehydration therapy; and maternal, newborn and child health) to provide performance‐based monetary incentives to volunteers rather than a fixed salary to conduct home visits (one per 250–300 households) and supported them with full‐time mentors (IYCF promoters) with one mentor per 10 volunteers. Volunteers received a cash incentive (totaling US$6 to US$8 per month on average) based on each eligible mother who practised the IYCF behaviours. This motivated volunteers to counsel almost every eligible woman in their respective areas and work on problem‐solving for changing behaviours rather than only giving messages.

### Programme implications of monitoring systems and small studies 2010–2014

Feedback from monitoring visits revealed that mothers often complained that they were unable to feed children because of ‘poor appetite’. This did not refer to ‘sick’ children only, or frequently sick children. A&T hypothesized that one reason for low appetite might be sub‐clinical enteropathic infections from contaminated complementary foods due to infrequent washing of hands with soap before feeding children (Islam *et al*. 2013). This led to the decision to conduct additional formative research on handwashing before child feeding, in order to provide more emphasis on the behaviour within the programme.

The monitoring system helped to highlight which programme sub‐districts were lagging in enlisting eligible households, in providing home visits and in improving IYCF indicators. A&T took special steps to ensure high coverage and quality counselling in home visits while scaling up:

- BRAC established 16 parallel training centres across all regions for meeting the demand for frontline workers and ongoing refresher training.
- Managers ensured full staffing of frontline workers in programme areas, and vacancies were filled.
- Supervision checklists were routinely collected and discussed.
- Mobile phone contacts were established between volunteers and salaried staff.

The focus of community mobilization shifted based on monitoring data of BRAC and A&T. For example, in the latter half of the programme period, fathers, religious leaders and doctors/local opinion leaders (combined group) were prioritized.

Results of knowledge, attitudes and practices surveys of doctors and their media habits in 2012 showed that doctors were not providing even basic counselling about child nutrition to mothers. They did not have the knowledge, skills or motivation for improving IYCF practices. There was some evidence that they may even have been reinforcing poor practices – for example, recommending breast milk substitutes for perceived insufficient milk and encouraging ‘soft foods’ that are low in energy and nutrient density for infants under 1 year old. The survey showed that a large proportion did not believe it was their responsibility to counsel mothers on IYCF. It also showed that doctors could be reached with high coverage through print media and TV.

Alive & Thrive decided IYCF counselling needed to be urgently mainstreamed within national health services. GOB leadership was important for introducing IYCF counselling during antenatal care and immunization contacts and also at sick child visits. Standards of care for maternities, training on how to initiate breastfeeding and special support for C‐section deliveries were needed. A&T provided a partnership grant to the Obstetrical and Gynecological Society of Bangladesh (OGSB) to support revisions to the pre‐service curriculum. The programme also launched a mass media campaign through inserts in newspapers for 20 months starting in October 2012.

### Programme implications from formative research on handwashing

The household survey on handwashing revealed that 95% of participants had soap in their homes but only 37% had soap present at the place for handwashing before food preparation (Nizame *et al*. 2013). Participants connected food contamination with the presence of dust/dirt and also with germs but did not think that hands could be a vehicle for germs if no dirt were visible. Most (60%) of surveyed respondents agreed with the statement that ‘the unavailability of soap and water near the cooking place was a physical barrier to handwashing before food preparation’.

Alive & Thrive tests introducing handwashing stations to households for use at the location of food preparation showed practices improved with both home‐made stations and ready‐made project‐provided stations. However, providing stations on a large scale was not sustainable (Biswas *et al*. 2014). The same study also showed that complementary feeding indicators improved alongside handwashing indicators when a single integrated package of interventions was implemented (Unicomb *et al*. 2013).

Alive & Thrive decided that home visits should include helping families to establish and maintain handwashing stations close to the place of child feeding rather than focusing on the message ‘wash your hands with soap’. Mass media and community mobilization were used to create an enabling environment by changing perceptions about using soap as the norm. Communication materials and a TV spot focused on convenience – establishing a handwashing close to the place of food preparation. A training session on handwashing stations was also inserted in the national IYCF training manual.

### Programme implications of the midline surveys in 2012 and 2013

The baseline survey in 2010 and early midline in 2012 showed that IYCF TV spots were seen by the same per cent of rural mothers in intensive and non‐intensive areas, and coverage was about 50%. Opportunistic media recall surveys in 2012 (attached to other A&T assessments) conducted in Sylhet (eastern region), Manikgonj (central region) and Dinajpur (northern region) also showed that a significant number of communities did not view TV because of electricity shortages and exclusion from services due to various reasons, and this was contributing to low TV viewership.

Alive & Thrive tested a supplementary communication strategy of exposing these communities to the same TV spots through mobile video shows and interactive Q&A sessions (conducted by rural marketing agencies) in order to reach a wide cross‐section of community members and opinion leaders in low‐electricity communities. The materials included A&T messages contained in *Meena* animated films and the seven TV spots being broadcast through national TV. Each material was played, and key messages were then discussed by trained facilitators. Difficulties in practising the actions were also discussed. A small feasibility trial in 19 randomly allocated low electricity villages confirmed that even a single round of such an approach would raise message recall and knowledge of correct practices. Based on this, the ‘media dark’ strategy was mainstreamed in the A&T programme.

The 2013 midline survey showed improvements in TV exposure above 2010 and 2012 levels in both A&T programme areas (Menon *et al*. 2014). In intensive areas, the improvement in 2013 was slightly greater where the media dark strategy was implemented for hard‐to‐reach and low‐electricity communities.

The 2012 midline survey of mothers with 6‐ to 23‐month‐old children showed only 45–50% coverage of home visits in targeted areas. From 2012 to 2014, coverage of home visits was consistently higher and increased over time in areas receiving more intensive inputs. Midline survey results in 2013 and the endline in 2014 found that the reach of BRAC frontline workers had risen to over 80% in A&T intensive areas.

At the 2013, midline five of the eight key IYCF indicators showed significant improvements over the 2010 baseline levels in both programme intensive and non‐intensive areas, and the impacts were greater in intensive areas. From 2010 at baseline to 2013, IYCF practices improved in intensive areas [double difference (DD) compared with non‐intensive areas] as follows: early initiation of breastfeeding from 63.5% to 91.9% (DD of 18.7), exclusive breastfeeding from 48.5% to 83.4% (DD of 25.0), minimum dietary diversity from 32.1 to 61.8 (DD of 26.5), minimum acceptable diet from 16.0 to 48.0 (DD of 24.6) and iron‐rich foods from 39.5 to 72.2 (DD of 20.9).

Problems in IYCF still remained, including not feeding during illness and use of sugary drinks and foods and salty snacks.

The processes that were associated with improvements in practices included high levels of exposure to TV spots on IYCF in both intensive and less intensive programme areas and, in intensive areas, higher frequency of home visits by trained IYCF workers (Saha *et al*. 2013). In A&T‐intensive areas, frontline workers were named more often as the primary sources of IYCF information than family members and neighbours – a change from the 2010 baseline survey.

## Discussion

### Ingredients for scale

In a review of scaling up innovations across development sectors, Linn (2012) concluded that among the essential ingredients of successful scale‐up are a commitment to scaling up from the start, systematically planned steps to achieve scale in the programme design process, agility to adapt the approach because successive stages of scale‐up require different inputs, the strategic use of data to manage these turning points and partnerships that are built for long‐term sustainability.

Perez‐Escamilla *et al*. (2012) found that facilitation of scale‐up of breastfeeding programmes in Africa, Asia, Latin America and the Caribbean involved multiple components that worked in close harmony. Evidence‐based advocacy was required for shifting political will to enact legislation and policies; human and financial resources were important for ensuring a large cadre of trained frontline providers to support mothers, and data that were fed back for making programme adjustments were valuable to ensuring quality during scale‐up. Victora *et al*. (2012) noted that successful scale‐up of maternal nutrition programmes required making the problem visible to decision makers; evidence‐based programme design and large‐scale implementation capacities were essential; partnerships among governmental and private (commercial and nonprofit) agencies facilitated the process; and scale‐up could be accelerated by generating demand within the target population.

In Bangladesh, A&T's process of using data from multiple sources to design and continually adjust strategies while rapidly scaling up a national programme was another ingredient that resulted in significant changes in IYCF practices. Selection of key practices for programme focus was a first important step. A&T focused on converting high awareness on breastfeeding to desirable practices and building a basic understanding about complementary feeding.

### Achieving behaviour change on a large scale

The use of a sound theoretical basis to design behaviour change interventions and engaging complementary channels to reach diverse audiences were also important. The programme employed a socio‐ecological model of behaviour change (McLeroy *et al*. 1988), addressing multiple factors found by the formative research studies (conducted in different regions of the country and among national thought leaders) to be major determinants of individual IYCF practices. The intervention strategy engaged various sources of influence on mothers through specifically tailored face‐to‐face contacts (through advocacy dialogue, community meetings/forums, home visits and clinic visits) and mass media (TV spots on father's support and print media plus TV spots for health providers). Religious leaders constituted a prime channel for reinforcing information within communities. Children were a secondary audience, reached through the *Meena* animated film series and in popular TV programmes, as part of a longer‐term strategy to educate the next generation and also as a potential route for delivering messages in the home.

A training programme for community volunteers and health workers was combined with supportive supervision, monthly meetings, quarterly refresher training, monitoring feedback and monetary incentives to both improve skills and incentivize performance aimed at solving problems and using meaningful motivations for mothers and families, not just completing scheduled home visits for delivering messages.

### Strategic planning for scale from the start

Alive & Thrive selected partners and existing platforms to scale up rapidly within and outside BRAC. In expanding small‐scale programmes, organizations may face challenges related to building management capacity (Cooley & Kohl 2006). In Bangladesh, however, A&T was able to utilize the structures and experience of BRAC with limited additional investment in field offices and orientation to multi‐channel communication strategies. Planning for scale involved setting up administrative systems within BRAC to meet the needs of multiple training sites, hiring additional staff in key posts and building capacity for recordkeeping and information flows focused on a few key indicators for monitoring.

Other scale‐up strategies included systematic development of an evidence‐based package of tools and government (or multiple stakeholders) branding of the package for broader ownership and uptake. The national IYCF communication plan framework and document developed by the IYCF Alliance with A&T and UNICEF support was also branded and distributed by the government and adopted by a range of stakeholders. A&T also made adoption convenient by providing seed money, timely technical support and free materials and training of trainers to government and NGOs.

### Facilitating adoption by multiple implementers

After launching the programme in 2010, A&T programme components were streamlined starting in 2012 as part of the transfer process to new implementing partners. For example, training of frontline workers was reduced from 5 to 3 days. Post‐training supervision checklists were reduced from over 25 items each for breastfeeding and complementary feeding to a single two‐sided sheet with 8–10 items for observing counselling content and technique. The number of separate community mobilization forums was reduced from 8 (religious leaders, youth/adolescents, school teachers, traditional birth attendants (TBAs), other NGO workers, village elite, local government leaders and village doctors) to 3 (doctors, combined group of local opinion leaders and fathers of children aged 6–23 months).

Programme scale and effectiveness increased over time because of monitoring and purposeful adjustment of strategies that led to new implementing partners. When the 2012 midline survey showed that mothers continued to consult doctors for IYCF difficulties, A&T increased its emphasis on reaching doctors, through both in‐service and pre‐service training. New communication channels were employed (newspaper inserts) to reach medical doctors; the national OGSB was selected as a strategic partner to lead the strengthening of pre‐service medical and nursing curricula.

The formation of the National IYCF Alliance of multiple stakeholders by A&T and UNICEF under GOB/IPHN facilitated expansion of the IYCF programme beyond BRAC to new implementers. The transfer of approaches, tools and skills nationwide was carried out through this central government‐led hub. Collaboration with UNICEF (GOB's primary external technical support agency) and advocacy with donors such as DFID and USAID helped lay the groundwork for sustainability. In the second year of implementation, the country's national nutrition programme (supported by multiple donors) was reorganized as the National Nutrition Service, and with A&T's inputs, IYCF became a top priority in plans for mainstreaming through government health and family planning services.

### Scale‐up processes and pathways

Scale‐up took place in several ways. Partnerships led to the geographic expansion of the A&T community model and tools within different BRAC programme divisions and outside BRAC (BRAC 2014). The government, UNICEF and the National IYCF Alliance were identified by A&T as key players in scaling up. Within the BRAC's EHC programme, the A&T programme expanded from two sub‐districts in 2009 to 22 in 2010; another 28 sub‐districts were added in 2011, for a total of 50 A&T‐supported BRAC/EHC sub‐districts (Haque *et al*. 2012). In 2014, the A&T package of community interventions was expanded to another 114 BRAC/EHC sub‐districts with funds from DFID. Other BRAC programmes, particularly for urban and rural Maternal Newborn and Child Health (MNCH), were expanded from one sub‐district in 2012 to a total of 94 sub‐districts in 14 districts in 2014. Expansion of non‐BRAC stakeholder programmes began in 2012 and continued until 2015. Two examples where A&T advocated for and later helped design the adaptation for new geographic areas and types of programme platforms are the DFID‐funded Livelihoods programmes in hard‐to‐reach and urban poor communities and the USAID‐supported Feed the Future programme.

To increase the commitment of new stakeholders to IYCF, A&T's advocacy component engaged national medical associations of paediatricians and Ob/Gyn practitioners, as well as donors and senior government officials. Journalists were invited to raise awareness by reporting on the risks of stunting and related IYCF government programmes and policies.

### Contextual factors

Successful scale‐up was facilitated in Bangladesh by contextual factors. Publicity around the global Scale Up Nutrition (SUN) movement movement stimulated involvement in IYCF by new donors. Different constituencies responded for different reasons. Government historically had progressive policies (e.g. 6 months of maternity leave and International Code of Marketing of Breast‐milk Substitutes) and strategies (National Strategy for IYCF and National Plan of Action) but stagnant indicators (NIPORT 2005; NIPORT 2009). Government also needed a quick replacement for the recently disbanded National Nutrition Programme. Breastfeeding was well accepted in the newborn child mortality community as a newborn and infant mortality‐reducing intervention. Among nutritionists, there was interest in lowering high levels of underweight, stunting and wasting in the country through the IYCF component. There was an interest in learning more about programme strategies to address child malnutrition, as several projects were working towards a goal of reducing malnutrition and A&T was the only one solely dedicated to improving IYCF practices. Donors such as DFID and medical associations such as OGSB wanted to engage in a strongly evidence‐based programme with high payoffs. Early results from the midline surveys by IFPRI provided credible evidence (from a rigorously designed external evaluation) of significant impact.

The National IYCF Alliance – comprising A&T, BRAC, UNICEF and the government as its nucleus plus over 20 other stakeholders – led to building up resources for scale and is now a formal structure of the government of Bangladesh.

### Challenges

Challenges faced during scale‐up included (1) lack of demand due to widespread gaps in understanding among communities and health workers of complementary feeding beyond introduction at 6–8 months; (2) lack of capacity, leadership and confidence including concerns among implementing partners that mainstreaming IYCF into existing programme platforms might not work because of work overload and lack of technical focus; (3) a limited number of experienced nutrition programme implementers with the necessary practical training; (4) unfamiliarity among implementers with WHO's IYCF indicators; (5) the need for training and supervising a large number of health care providers and community volunteers in the labour‐intensive interpersonal component; (6) a substantial number of the population residing in ‘media dark’ areas; and (7) one in four households facing economic constraints. Donors were reluctant to make adequate financial commitments without firm evidence of results from the endline survey in 2014.

## Conclusion

Key to scale‐up of A&T's IYCF interventions in Bangladesh included early evidence that behaviour change interventions were working, partnership with a strong community‐based NGO, the formation of alliances with like‐minded stakeholders, availability of funds and technical support from multiple donors, feasible programme implementation options, well‐defined interventions and indicators, and streamlined processes and tools to aid implementation. Formation of synergistic partnerships for sustainability and scale – particularly the IYCF Alliance, including governmental, non‐governmental and donor organizations – ensured complementary resources to support IYCF activities.

## Source of funding

Alive & Thrive is funded by the Bill & Melinda Gates Foundation and the governments of Canada and Ireland.

## Conflicts of interest

The authors declare that they have no conflicts of interest.

## Contributions

TS, JB, RS and AJ contributed to writing different sections. SR, RH and KA provided concepts, interpretation and facts and figures for the document.
